# Effect of Air Abrasion on the Number of Particles Embedded in Zironia

**DOI:** 10.3390/ma11020259

**Published:** 2018-02-08

**Authors:** Beata Śmielak, Leszek Klimek

**Affiliations:** 1 Department of Dental Prosthetics, Medical University of Lodz, Pomorska Str. 251, 92-213 Lodz, Poland; 2 Department of Dental Technology, Medical University of Lodz, Pomorska Str. 251, 92-213 Lodz, Poland; 3 Department of Materials Research, Institute of Materials Science and Engineering, University of Technology, Stefanowskiego Str. 1/15, 90-924 Lodz, Poland; leszek.klimek@umed.lodz.pl

**Keywords:** air abrasion, abrasive particles, zirconia

## Abstract

**Background**: Determination of the number of abrasive particles embedded in the zirconia surface after variable parameters of treatment. Methods: One hundred thirty cylindrical disks made from zirconia were divided into 7 groups (*n* = 10): one control and six test groups treated by air abrasion using Al_2_O_3_ or SiC with grain sizes: 60, 110, 250 μm with a working pressure of 0.2 or 0.35 MPa. The SEM images were observed in BSE and BSE 3D. The chemical composition was determined by means of X-ray microanalysis with EDS. The surface was determined by quantitative metallography methods. Surfaces (%) depending on the particle type were compared using the Mann-Whitney test, depending on the pressure were compared using the Mann-Whitney test, and depending on the grain size were compared using the Kruskal-Wallis test as well as the Jonckheere-Terpstra trend test as well as the Dunn post-hoc testA probability. Value of *p* < 0.05 was deemed significant, while a *p*-value of *p* < 0.01 was regarded as highly significant. Results: After blasting aluminium and silicon particles were embedded in zirconia surface. When blasted with Al_2_O_3_, the average amount of embedded grain was higher, while in the case of SiC. Highly significant differences were observed in the surface share of the abrasive depending on the grain size. At a pressure of 0.20 MPa the quantity of embedded abrasive amounted to 6.63, and at the pressure of 0.35 MPa rose to 7.17. Most particles of abrasive material became embedded when sandblasting with grain size 60 μm grain. No significant difference was observed in the surface share of the abrasive depending on the pressure. Conclusion: The quantity of embedded abrasive depends on its type and grain size, and the pressure applied.

## 1. Introduction

Air-borne particle abrasion is widely used in dental technology. Its main function is to remove excess material, provide shape to the treated elements and create a suitable surface in dentures. This form of treatment utilises the kinetic energy of abrasive grains in a stream of compressed gas (usually air). The accelerated abrasive grains strike the surface of the treated element and this process results in abrasive grain particles micro-cutting the substrate, which, as a consequence, results in loss of material. This process is often referred to as abrasion and the treatment is known as abrasive blasting.

Abrasive blasting is already applied in the initial stages of preparing prosthetic restorations with the aim of cleaning dental alloy castings. It is also a routine method for preparing the surface of metal alloys prior to ceramic firing. Its purpose is to expand the surface and change such physicochemical properties as, e.g., the electrostatic potential and free surface energy [1]. Changes in the surface lead to an increase in the bonding strength between the metal and ceramic through the creation of surface roughness by mechanical means, which later allows liquid ceramic to attach and penetrate. Abrasive blasting treatment also improves bonding strength by removing weakly attached overhangs and metal flakes formed during the grinding process, which ensures better anchoring and increases wettability [2,3]. Moreover, in the case of certain connection systems, e.g., titanium-ceramic, this method is practically the only possible way of increasing bond strength owing to titanium’s properties.

The use of air abrasion to treat zirconium dioxide (3 TPZ-Y) surfaces is a highly debatable issue. Its extreme hardness (12.17–13.7 GPa) impedes surface expansion and makes it difficult to prepare the surface properly for ceramic firing [4,5,6]. Many scientific studies deny that air abrasion has any significant effect on improving bonding with veneering ceramic, regardless of the grain size [7,8]. Moreover, other studies demonstrate the negative influence of air abrasion on the structure of zirconium dioxide [9]. However, the majority of authors show that air abrasion improves the quality of the bond with veneering ceramic and should be recommended [10,11,12,13].

Air abrasion is known to cause an unfavourable tetragonal-to-monoclinic phase transformation in material, which consequently results in surface tension [14]. In the opinion of certain authors, this transformation makes the material more susceptible to damage, which in turn may cause surface ageing as the grain crumbles, microcracks appear, and the strength of the material is reduced [15,16,17]. According to other researchers, the thickness of the layer in which the transformation occurs is insufficient to cause microcracking and reduce flexural strength [18].

What is certain is that during blasting abrasive particles with high kinetic energy become embedded in the treated material. It is not entirely known whether this phenomenon has negative or positive effects. The particles left in the material reduce the surface smoothness [19,20]. When porcelain is fired on air-borne particles abraded surfaces the role of embedded particles is not entirely clear. On the one hand, they expand the treated surface, which may improve the quality of the bond. On the other, they are accompanied by phase destabilisation and the breaking of the grains, and they may also initiate fracturing of ceramic when prosthetic restorations are worn [21]. Also not without importance is the possibility that embedded abrasive grains may react with the fired ceramic. It is thus of key importance to determine the quantity of grains that become embedded as different blasting parameters are applied. This may be important for establishing the appropriate blasting parameters for zirconium dioxide. This is because, in spite of the claims of many studies, this bond is still the weakest point of prosthetic restorations and results in chipping and fracturing.

The objective of this study was to examine the influence of selected abrasive blasting parameters on the quantity of Al_2_O_3_ and SiC abrasives embedded on the surface of zirconium dioxide (3 TPZ-Y).

## 2. Materials and Methods

A total of 130 cylindrical specimens of 3Y-TZP (Ceramill Zi; Amann Girrbach AG, Koblach, Austria) were sintered in a furnace (Ceramill Therm; Amann Girrbach AG) by using a universal program (8 °C per minute from 200 °C to 1450 °C, 2 h at a fixed temperature of 1450 °C, and the correct cooling time). The sintering process lasted approximately 10 h. Material shrinkage amounted to approximately 21%. After sintering, the specimens had a diameter of 9 mm and height of 5 mm.

To make the surface uniform before airborne-particle abrasion, the disks were ground on a rotary grinder (Metasinex; Metasinex Row, Poland) with SiC abrasive paper with a grit size of 220, 400, 600, and 800 under water cooling, washed in an ultrasonic washer (Quantrex 90 WT, L&R Manufacturing, Inc., Kearny, NJ. USA) in ethyl alcohol for 10 min and dried with compressed air (Tornado 4, Durr Dental AG, Bietigheim-Bissingen, Germany). An oil-free air compressor with a particulate filter was used.

Ten samples were left after grinding to serve as a control group. The rest of the specimens were treated with an airborne-particle abrasion process (Mikroblast Duo; Prodento-Optimed, Warsaw, Poland) using Aluminum oxide (Al_2_O_3_) or silicon carbide (SiC). A fixed angle of 45 degrees and a distance of 10 mm from the airborne-particle abrasion nozzle (Mikroblast Duo; Prodento-Optimed) were chosen for further experiments. The abrasion time of the specimens was established at 10 s.

The variable parameters were as follows:

- size of grain: 60, 110, 250 μm,
- working pressure: 0.2, 0.35 MPa.

**Figure 1:**
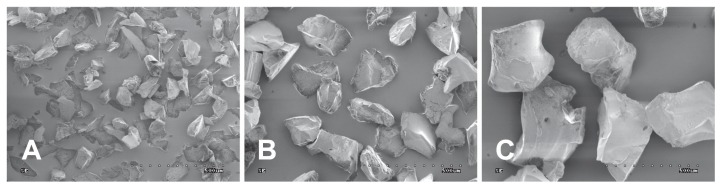
Selected of SE images of aluminium oxide grains. (**A**) 60 μm; (**B**) 110 μm; (**C**) 250 μm.

Figure 1 shows images of aluminium oxide grains. Grains marked 60 μm are typically between 53 and 75 μm in size. Grains marked 110 μm are typically between 106 and 125 μm in size. Grains marked 250 μm are typically between 238 and 275 μm in size.

**Figure 2:**
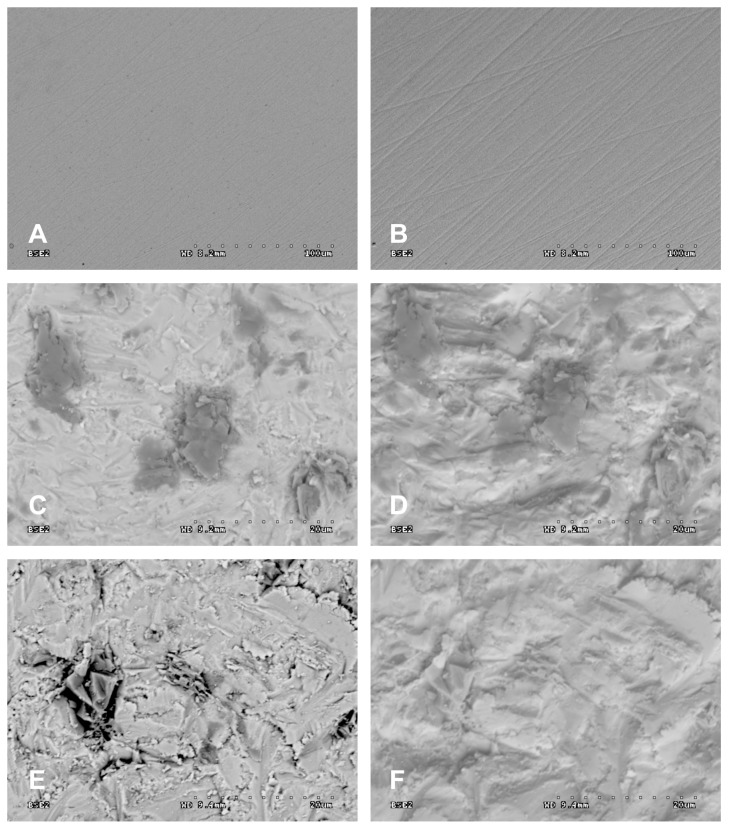
Results of SEM examination (original magnification 500×) obtained with BSE and BSE 3D on zirconia surfaces: (**A**) after grinding (BSE); (**B**) after grinding (BSE 3D); (**C**) after blasting with Al_2_O_3_110 μm particles under pressure of 0.2 MPa (BSE); (**D**) after blasting with Al_2_O_3_ 110 μm particles under pressure of 0.2 MPa (BSE 3D); (**E**) after blasting with SiC 110 μm particles under pressure of 0.2 MPa (BSE); (**F**) after blasting with SiC 110 μm particles under pressure of 0.2 MPa (BSE 3D).

After abrasive blasting the samples were cleaned with pressurised steam, washed in deionized water in an ultrasonic washer for 8 min, after which they were dried with compressed air. The specimens were examined with an electron scanning microscope (SEM S-3000N; Hitachi High-Technologies Corp, Tokyo, Japan). Depending on the type of registered signal emitted by the specimen (after being stimulated by electron beams), 2 types of images were registered in SE secondary electrons (surface topography) and retrospectively in backscattered BSE electrons (so-called material contrast). Figure 2 shows selected SEM images obtained with back-scattered electrons (BSE) (“material contrast”) and BSE 3D (surface topography) after grinding and blasting with Al_2_O_3_ and SiC 110 μm particles under pressure of 0.2 MPa.

**Figure 3:**
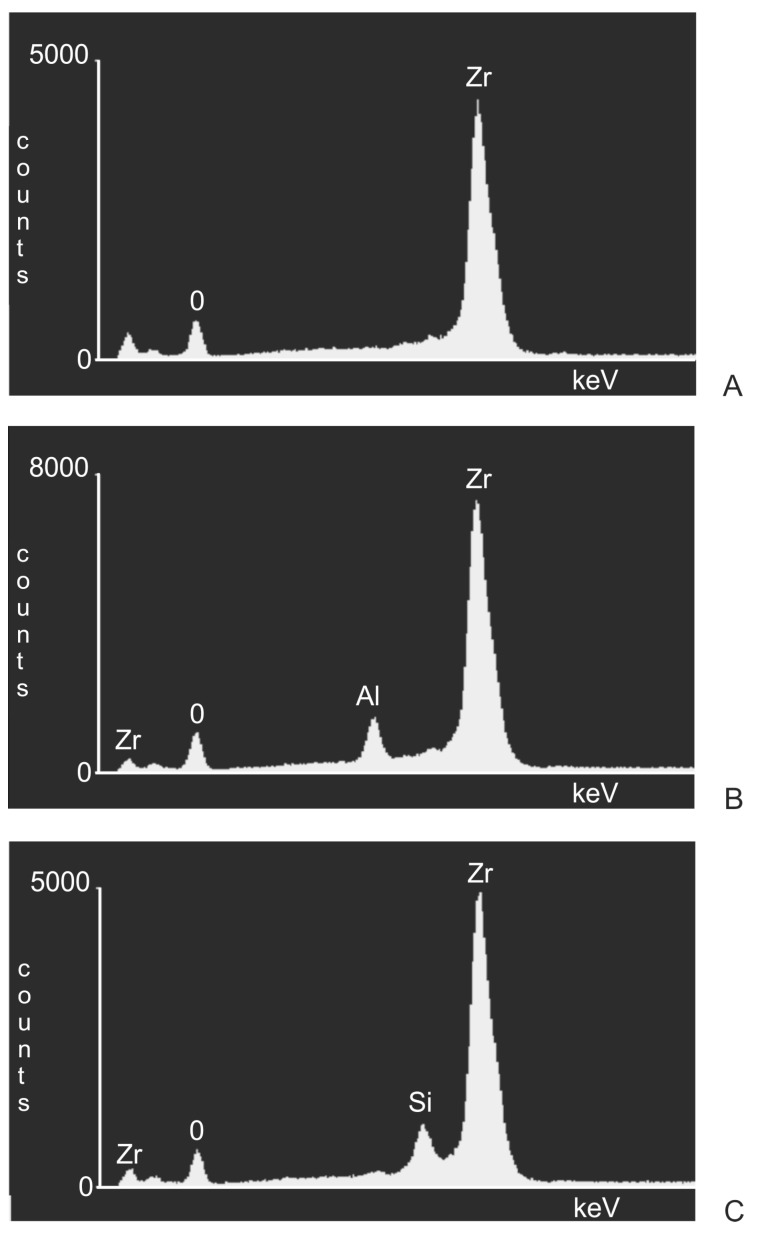
EDS spectrograms of zirconia surfaces: (**A**) after grinding; (**B**) after blasting with Al_2_O_3_; (**C**) after blasting with SiC.

Dark areas were visible on the surfaces of the samples following abrasive blasting, indicating differences in the chemical composition of these areas in relation to the treated substrate. The chemical composition of the samples was determined on the basis of an X-ray microanalysis with an energy dispersive spectrometry EDS, using a microanalysis attachment produced by Thermo Noran in combination with SEM. Sample EDS spectrograms are shown in Figure 3.

**Figure 4:**
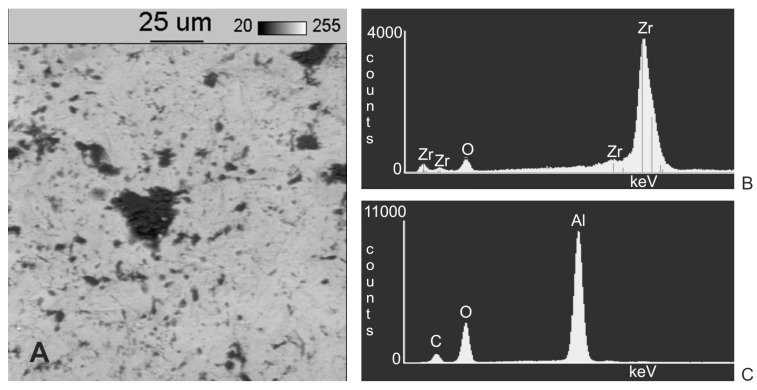
(**A**) Results of SEM examination (original magnification 800×) on zirconia surfaces after blasting with Al_2_O_3_; (**B**) EDS analysis of a light area; (**C**) EDS analysis of a dark area.

**Figure 5:**
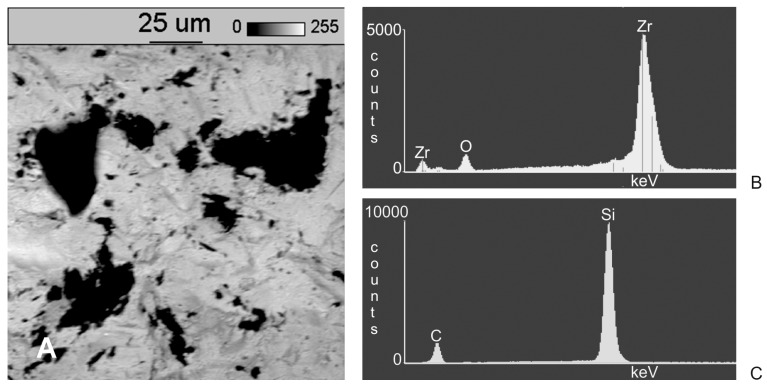
(**A**) Results of SEM examination (original magnification 800×) on zirconia surfaces after blasting with SiC; (**B**) EDS analysis of a light area; (**C**) EDS analysis of a dark area.

**Figure 6:**
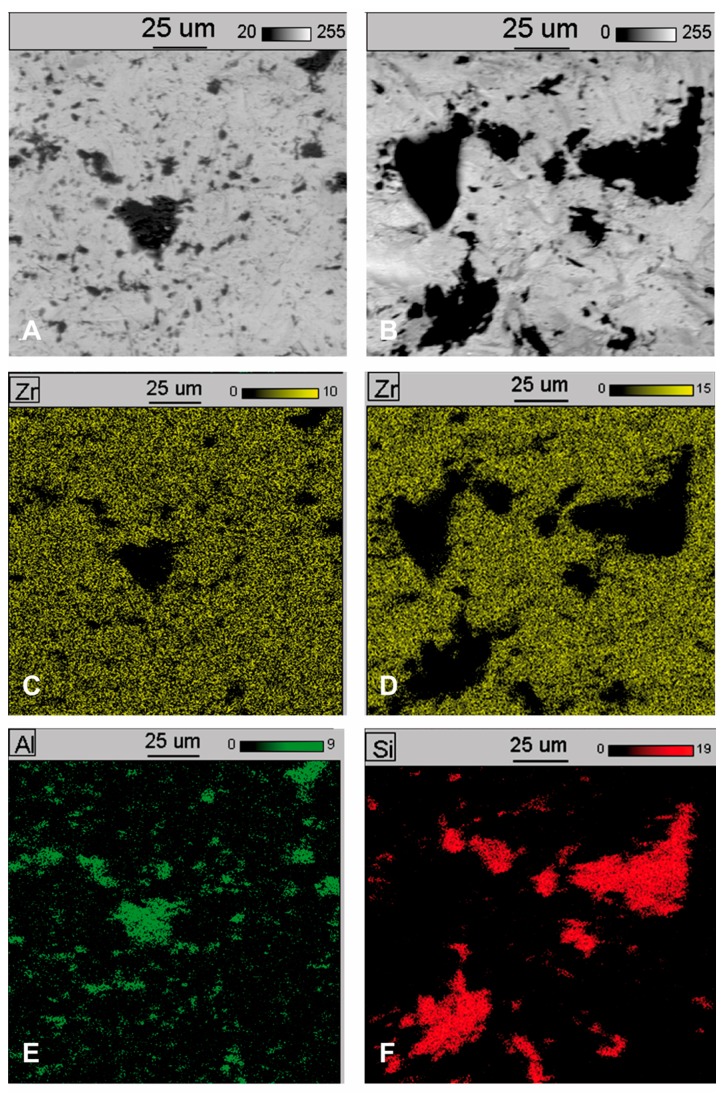
Results SEM examination (original magnification 800×) and distribution of elements on sample surfaces: (**A**) SEM examination on zirconia surface after blasting with Al_2_O_3_; (**B**) SEM examination on zirconia surface after blasting with SiC; (**C**,**D**) surface distribution of zirconium; (**E**) surface distribution of aluminium; (**F**) surface distribution of silicon.

EDS spectrograms of treated samples showed, besides signals of zirconia (Zr) and oxygen (O) coming from the base of the samples, signals from additional elements. An additional signal from aluminium in a sample blasted with aluminium oxide (Al_2_O_3_) as well as from silicon in a sample blasted with silicon carbide (SiC) indicates they originate in the abrasive material. To identify and determine which areas (dark or light) observed under a microscope originate from abrasive material, additional observations were made of samples under SEM at 800× magnification, together with spectrograms of dark and light areas. In addition, a surface distribution map was prepared of elements in the observed areas. Sample SEM images following Al_2_O_3_ and SiC treatment are presented in Figure 4A and Figure 5A as well as an EDS analysis are presented in Figure 4B, Figure 5B,C and Figure 6C. The surface distribution maps of elements are set out in Figure 6.

The dark elements visible in the microscope image and shown in Figure 4A contain aluminium, while the dark elements presented in Figure 5A contain silicon.

This shows unequivocally that they are embedded abrasive particles. The analysis confirmed the presence of Al in samples following blasting with Al_2_O_3_ and Si following blasting with SiC in relation to the original sample (without blasting).

**Figure 7:**
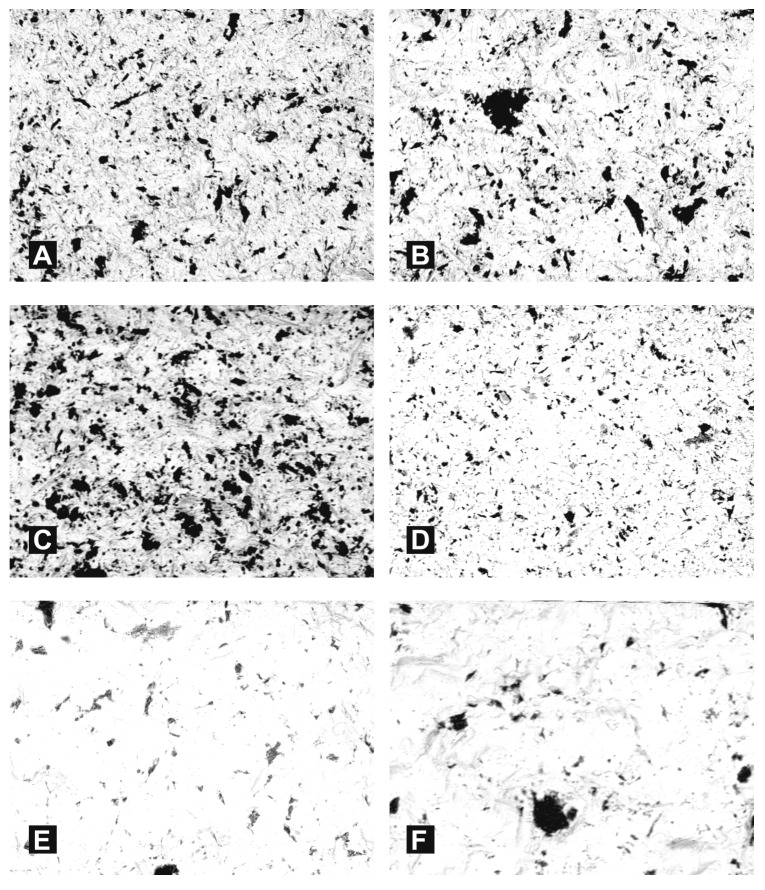
Selected results of SEM examination (original magnification 500×) on zirconia surfaces (backscattered electrons BSE): after blasting with Al_2_O_3_ with pressure 0.35 MPa and the following grain sizes: (**A**) 60 μm; (**B**) 110 μm; (**C**) 250 μm after blasting with SiC with pressure 0.35 MPa and the following grain sizes: (**D**) 60 μm; (**E**) 110 μm; (**F**) 250 μm.

Then, with the aim of showing and determining the number of grains embedded in the treated surfaces, 10 images were taken at each of a number of randomly selected sites based on SEM microscope images with back-scattered electrons (material contrast). Exemplary images of samples are presented in Figure 7.

**Figure 8:**
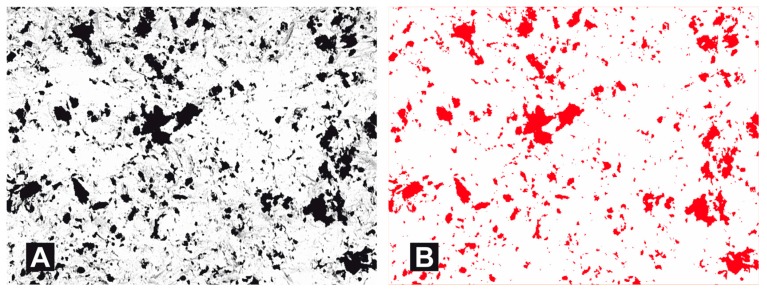
Binarization of microscopy image (**A**) original image (**B**) Image after binarization (for calculation purposes).

Then, the surface coverage of the abrasive material particles was determined with quantitative metallography methods using Metillo software [22,23,24,25,26,27]. The process was as follows:

1. Microscope image loaded into Metillo programme (Figure 8A).
2. Shadow correction.
3. Normalisation of grey level histogram (Figure 8B).
4. Manual binarization of image.
5. Calculation of surface share–in percentage terms–of dark (red) areas, as a share of abrasive elements embedded in the surface of a sample.

### Statistical Analyses

Statistical analyses of the results were conducted using PQStat statistical software version 1.6.4.122. Surfaces [%] depending on the particle type were compared using the Mann-Whitney test.

Surfaces [%] depending on the pressure were compared using the Mann-Whitney test.

Surfaces [%] depending on the grain size were compared using the Kruskal-Wallis test as well as the Jonckheere-Terpstra trend test as well as the Dunn post-hoc testA probability value of *p* < 0.05 was deemed significant, while a *p*-value of *p* < 0.01 was regarded as highly significant.

## 3. Results

**Figure 9:**
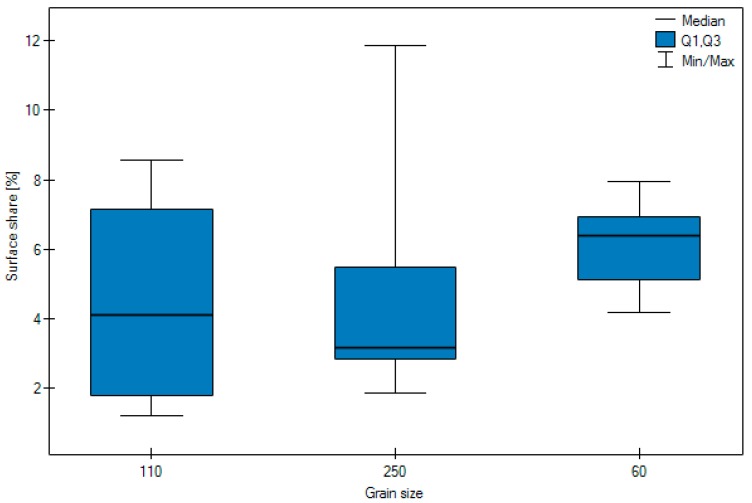
Graphic form of surface share of blasting depending on type of particle, pressure and grain size.

**Figure 10:**
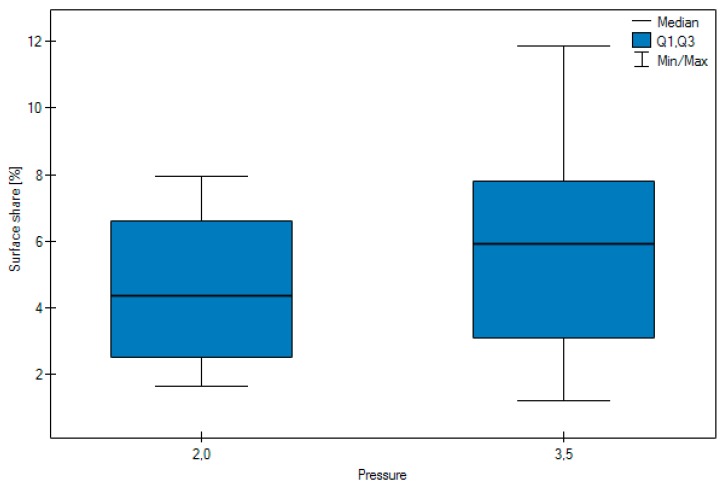
Surface share of abrasion, according to pressure.

**Figure 11:**
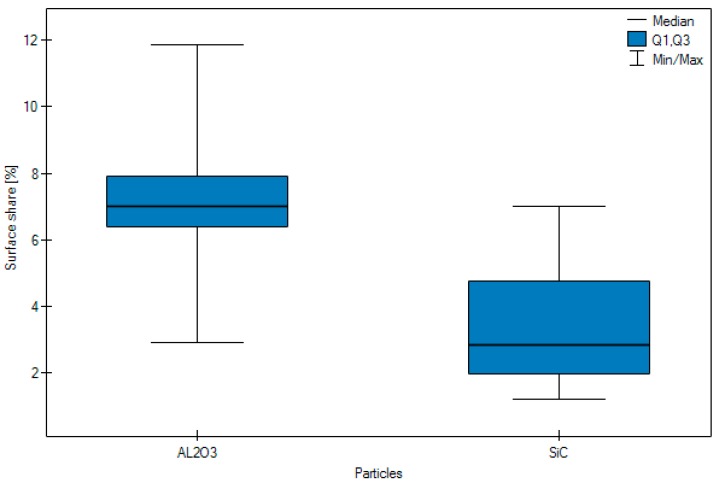
Surface share of abrasion according to grain size.

The results of the study, which calculated the number of embedded grains, are presented in Table 1 and Table 2. Figure 9, Figure 10 and Figure 11 show its in graphic form.

Highly significant (*p* < 0.0001) differences were observed in the surface share of an n abrasive, depending on the particle type. In the case of Al_2_O_3_ the results were in general higher than in the case of SiC.

No significant difference was observed in the surface share of the abrasive (*p* = 0.0509) depending on the pressure.

Highly significant differences were observed in the surface share of the abrasive (*p* = 0.0073) depending on the grain size. The Jonckheere-Terpstra trend test indicates a highly significant (Z = 3.5418, *p* = 0.0004) trend, i.e., the smaller the grain the higher the surface share of the abrasive. In the post-hoc analysis the results for the size 60 grain differ significantly (*p* < 0.05) from the results for grain sizes 110 and 250 μm, between which there no significant differences (*p* < 0.05).

Figure 9 surface share of abrasion according to particle type.

As the analysis shows, the conclusions are the same as in the case of Dunn’s test.

The analysed results revealed highly significant differences, depending on the abrasive type. In the case of Al_2_O_3_ the arithmetic mean of embedded grain from blasting was 7.10, while the average arithmetic mean in the case of SiC was 3.29.

The size of the grain was another highly significant factor impacting on the results obtained. The average amount of embedded abrasive in the case of a grain 250 μm in size is 4.99, compared with 4.51 recorded for a 110 μm grain, and 6.08 for a 60 μm grain. The amount of embedded 60 μm grain is significantly higher than when the material is blasted with 110 μm grain or 250 μm grain μm. No significant differences were observed with regard to the amount of pressure.

No significant difference was observed in the surface share of the abrasive (*p* = 0.0509) depending on the pressure.

## 4. Discussion

Microscopy observations (in back-scattered electrons) of sample surfaces following abrasive blasting treatment reveal the presence of a phase diverging from the 3TPZ-Y substrate. An exemplary analysis of the chemical composition of sample surfaces (Figure 4A and Figure 5A) performed under a scanning microscope demonstrated that particles of abrasive material–aluminium oxide or silicon carbide–remain on zirconia surfaces following abrasive blasting treatment.

Microscopic analysis already clearly reveals that—when comparing the surfaces of samples treated with grain of identical size (Figure 2)—the influence that the kind of abrasion particle and its gradation and applied pressure have on the state of the surface. More embedded abrasives were observed following treatment with Al_2_O_3_ grain than SiC treatment. This may be a consequence of differences in their hardness (Al_2_O_3_ 14–18 GPa, SiC 18–25 GPa). Perhaps because it is harder and more fragile SiC grains are more susceptible to crumbling on impact with the treated surface and thus fewer become embedded. This may especially be the case with small particles. The highest quantity of AI embedded in the surface of zirconium dioxide occurred with grains 60 μm in size.

The larger grains have themselves a larger surface area, i.e., the same numer of embedded grains should give a greater surface share, but there is still the possibility of grains crumbling and larger grains have a greater tendency to crumble. Besides this, larger graines could also be more weakly embedded in the surface, and as a consequence, due to their very weak connection, they could become detached during cleaning. This is probably the reason why of the three granularities tested we achieved the maximum with the 60 μm grain. This is confirmed by earlier studies [28].

An analysis of the quantity of embedded abrasive particles reveals the existence of a relationship between the type of grain and its size, but no between the amount of pressure applied. No significant difference was observed in the surface share of the abrasive depending on the pressure

In summing up the study it should be pointed out that during the course of the blasting treatment abrasive particles became embedded in the treated surface. Embedded elements may have a positive or negative impact on the treated surface of zirconium dioxide as well as on the quality of the bond with the veneering ceramic.

## 5. Conclusions

1. Following abrasive blasting treatment aluminium oxide and silicon particles were observed embedded in the treated surface of the zirconia.
2. A larger quantity of embedded abrasive was observed after blasting with Al_2_O_3_ than after blasting with SiC.
3. Most particles of abrasive material became embedded when blasting with size 110 grain.
4. No significant difference was observed in the surface share of the abrasive depending on the pressure.

We declare that the research is disclosed all conflicts interest statement, or explicitly state that there are none.

## Figures and Tables

**Table 1:** Surface share of abrasive depending on type of particle, pressure and size of grain-Dunn’s test.

Descriptive Statistics	Particles		Pressure (Mpa)		Grain Size (μm)		
	Al_2_O_3_	SiC	0.2	0.35	250	110	60
Arithmetic mean	7.10	3.29	4.56	5.83	4.99	4.51	6.085
Median	7.02	2.84	4.35	5.91	3.17	4.10	6.385
Standard deviation	2.34	1.59	2.06	3.21	3.62	2.78	1.0344
Minimum	2.93	1.21	1.63	1.21	1.86	1.21	4.19
Maximum	11.86	7.01	7.94	11.86	11.86	8.57	7.94
Lower Quartile < 0	6.38	1.96	2.52	3.08	2.84	1.80	5.1175
Upper quartile	7.91	4.75	6.61	7.79	5.49	7.14	6.945
Test statistics	Z = 7.9545		Z = 1.9525		H = 9.8445		
*p*	*p* < 0.0001		*p* = 0.0509		*p* = 0.0073		
Dunn’s test (post-hoc)	Grain size (μm)		250			0.7237	0.0116
			110		0.7237		0.0040
			60		0.0116	0.0040	

**Table 2:** Surface share of abrasive depending on type of particle, pressure and size of grain: Dunn’s test with Bonferroni and, Sidak corrections as well as the Conover-Iman test.

Post-Hoc	Grain Size (μm)	Grain Size (μm)		
		250	110	60
**Dunn-Bonferroni correction (post-hoc)**	250		10,000	0.0349
	110	10,000		0.0121
	60	0.0349	0.0121	
**Dunn-Sidak correction (post-hoc)**	250		0.9789	0.0345
	110	0.9789		0.0120
	60	0.0345	0.0120	
**Conover-Iman test (post-hoc)**	250		0.7150	0.0102
	110	0.7150		0.0035
	60	0.0102	0.0035	
